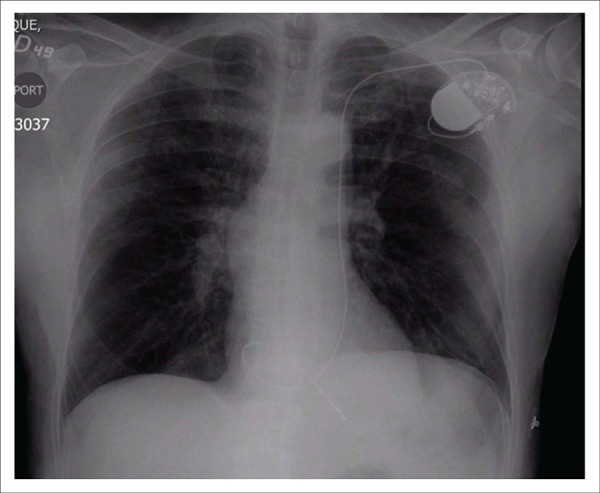# Persistent Left Superior Vena Cava in Permanent Pacemaker Implantation

**DOI:** 10.5935/abc.20150116

**Published:** 2015-10

**Authors:** Jerson Hernando Quitián, José Julian Carvajal, Mariana Soto, Guillermo Mora

**Affiliations:** 1 Hospital Universitario Fundación Santa Fe de Bogotá, Bogotá – Colombia; 2 Universidad de los Andes, Bogotá – Colombia; 3 Universidad Nacional de Colombia, Bogotá – Colombia

**Keywords:** Cardiopatias Congênitas, Veia Cava Superior, Marca - Passo Artificial

This was an 84-year-old male patient, with worsening functional class from NYHA III/IV to IV/IV and palpitations. No syncope. Physical examination revealed bradycardia, the rest was uneventful. Electrocardiogram showed Mobitz II atrioventricular block. Patient was scheduled for permanent pacemaker implantation.

Subclavian vein access was performed via direct puncture. The guide wire was advanced, entered the subclavian vein and descended parallel to the spine without crossing over to the right side. Subsequently, the guide wire traversed the coronary sinus and ended in the right atrium. Persistent left superior vena cava was diagnosed. During fluoroscopic observation, another feature that aids in the diagnosis is left paravertebral shadow above the aortic bow. The electrode was initially introduced with a straight guide reaching into the right atrium (RA). Afterwards, the straight guide was replaced by a conventional J guide and the electrode was pushed towards the anterolateral wall of the RA. The electrode tip was thus lying against the tricuspid valve. Subsequently, the guide was withdrawn 3 cm. The guide withdrawal, without moving the electrode, is associated with the passage of the electrode through the tricuspid valve. The electrode was advanced and finally the active fixation mechanism was deployed^1^.

## Figures and Tables

**Figure:**